# Response by Tariq to “Association Between Proton Pump Inhibitor Use and Short-term Postoperative Complications Following Abdominoplasty: A Multi-Institutional Cohort Study” by Wolf et al

**DOI:** 10.1093/asjof/ojag170

**Published:** 2026-09-05

**Authors:** Muhammad Ifrahim Tariq

I read with keen interest the recently published article by Wolf et al.^1^ The authors reported a higher risk of short-term surgical-site complications among patients with documented preoperative proton pump inhibitor (PPI) prescriptions than among matched H2-blocker users. The findings emphasize a potentially important issue in preoperative medication management.

The authors classified exposure as any documented prescription of PPIs in the year before abdominoplasty. Although they acknowledged a lack of data on medication adherence, they did not report data on recency, duration of use, and intermittent vs continuous exposure. A short-course PPI prescription issued 9 months before surgery and one issued immediately before surgery might be analyzed together within the same category. This heterogeneity may cause exposure misclassification and obscure, which pattern of PPI use was associated with observed outcomes. If the authors could clarify whether the data on prescription dates and duration were available, then a sensitivity analysis stratified by exposure recency and cumulative treatment duration could strengthen the interpretation of the findings. This is an important methodological consideration, as the proposed mechanisms involving dysbiosis may vary according to the duration of PPI exposure.^2^

The second methodological concern relates to assessment of post-matching covariate balance. Standardized mean differences (SMDs) are preferred over *P*-values because they are less influenced by sample size. The calculated SMD for White race was approximately 0.13, suggesting a residual imbalance. Therefore, reporting SMDs for all matched covariates would provide a more robust assessment of post-matching balance and allow readers to better judge the adequacy of the matching procedure.^3^

Prospective studies should evaluate whether the proximity of PPI use to surgery and cumulative duration are associated with postoperative complications and measurable gut microbiome changes, which may clarify the proposed dysbiosis-related mechanism. I appreciate the authors’ contribution and hope these methodological considerations strengthen the interpretation of their findings.
